# Factors Influencing the Maintenance of Public Health Behaviors After an Epidemic: Cross-Sectional Study

**DOI:** 10.2196/66535

**Published:** 2025-07-23

**Authors:** Xingmin Wang, Hongli Yan, Lushaobo Shi, Ting Li, Yi Xia, Dong Wang

**Affiliations:** 1 School of Public Health, Southern Medical University, Guangzhou, China; 2 School of Health Management, Southern Medical University, No. 1023 Shatai Road, Guangzhou, 510515, China, 86 02061647576; 3 Key Laboratory of Philosophy and Social Sciences of Guangdong Higher Education Institutions for Health Policies Research and Evaluation, Guangzhou, China; 4 Research Base for Development of Public Health Service System of Guangzhou, Guangzhou, China; 5 School of Humanities and Management, Guangdong Medical University, Dongguan, China; 6 Center for Faculty Development and Research, Guangzhou Medical University, Guangzhou, China

**Keywords:** health behaviors, maintenance, Multi-Theory Model, Protective Action Decision Model, influencing factors

## Abstract

**Background:**

The maintenance of public health behaviors stands as a critical issue within the realm of public health. COVID-19, a major global emergency, has profoundly impacted the sustainability of public health behaviors. However, there is currently a gap of empirical studies examining the status and influencing mechanisms of public health behavior maintenance after the pandemic, especially those adopting a multifactorial and integrated approach.

**Objective:**

This study aims to investigate the current status of public health behavior maintenance in China after the COVID-19 pandemic. It integrates the complementary advantages of the Multi-Theory Model (MTM) and the Protective Action Decision Model (PADM) to conduct a comprehensive analysis of the multi-factorial mechanisms influencing the maintenance of public health behaviors. The findings are expected to provide empirical evidence and strategic recommendations for the formulation of public health policies and the promotion of national health.

**Methods:**

An integrated model was developed based on the MTM and the PADM. Data were collected from the Chinese public between October and November 2023 via the online survey panel Sojump (Changsha Ranxing Information Technology Co., Ltd.). The questionnaire included items on health behavior maintenance, variables from the MTM and the PADM, sociodemographic and personal disease, and health characteristics. Univariate analysis, correlation analysis, multivariate regression analysis, and structural equation modeling were performed to explore the determinants of health behavior maintenance.

**Results:**

This study collected 1216 valid samples, including 726 females and 490 males, with an average age of 27.38 (SD 8.52) years, and most of them had been infected with COVID-19 at least once (1054/1216, 86.68%). The public maintenance of health behaviors was at a fairly low level (Mean 2.88, SD 0.45). Multivariate regression analysis revealed that those with high monthly incomes, married individuals, and who were more concerned about their health after the COVID-19 pandemic had higher levels of health behavior maintenance. These variables, along with others from the MTM and the PADM, accounted for 45.5% of the variance in health behavior maintenance. Structural equation modeling indicated that efficacy perception had the most significant positive influence on health behavior maintenance (*β*=.386, *P*<.001), followed by emotional transformation and practical changes (both *β*=.213, *P*<.001). Risk perception had a slightly negative effect on health behavior maintenance (*β*=−.099, *P*=.013). Variables such as social cues, warning messages, and information sources also indirectly influenced the public maintenance of health behaviors.

**Conclusions:**

This study indicates a slight decline in public health behavior maintenance following the COVID-19 pandemic, and our analysis has explored some of its influencing factors. Attention should be given to broadening information channels and appropriately explaining the risks of unhealthy behaviors. In addition, integrating external support and bolstering the public’s efficacy in maintaining health behaviors can promote sustainable healthy practices.

## Introduction

Health behaviors have a direct impact on an individual’s health status and are crucial for maintaining health and prolonging life. Gochman defines health behaviors as patterns, actions, and habits related to health maintenance, recovery, and improvement [1]. However, many health-promoting behaviors need to be practiced consistently over time to significantly affect health [2]. This leads to the important concept of health behavior maintenance, which refers to the sustained adoption of health behaviors to maintain overall health and well-being [3]. Engaging in long-term health behaviors, such as regular physical activity and a healthy diet, can greatly reduce the risk of severe acute infectious diseases and chronic noncommunicable conditions, including coronary heart disease, type 2 diabetes mellitus, and cancer [4,5]. Large-scale and sustained health behavior practices are vital as they help mitigate future pandemic risks and enhance preparedness [6].

The COVID-19 pandemic, as a global public health emergency, has exerted significant changes in public health awareness, behavioral patterns, and social support mechanisms, impacting the maintenance of public health behaviors. Studies reveal a divergence of health behaviors caused by the pandemic: some individuals persistently adopt protective measures, such as mask-wearing, due to heightened risk perception [7], while others exhibit behavioral relaxation attributed to “pandemic fatigue” or socioeconomic pressures [8]. Psychological traits, such as conspiracy beliefs, have been found to significantly influence compliance with protective behaviors [9], whereas social trust and cultural values, such as collectivism, regulate the sustainability of behaviors through group identity [10]. Although existing studies in China have addressed the impact of pandemic-related knowledge and anxiety on health behaviors [11,12], and there have also been studies on changes in health behaviors before and during the pandemic in European countries [13], there remains a notable gap in theoretical exploration regarding the dynamic mechanisms of health behavior maintenance and multifactorial interactions.

Numerous studies have explored health behaviors, but most existing studies mainly focus on the static characteristics of health behaviors rather than their continuity [14]. Current studies often concentrate on changes in specific types of health behaviors, such as dietary habits or disease prevention measures, with less attention given to integrating a variety of health-related behaviors. In addition, while psychological and cognitive factors, such as risk perception and efficacy perception, have been emphasized, there has been insufficient comprehensive analysis that incorporates both internal and external factors, such as health-related message and peer support [15]. Based on theories of health behavior change, some studies have been conducted on health education and promotion interventions for patients with chronic disease [16]. Although these studies have identified factors related to the maintenance of health behaviors within the context of interventions, whether health behaviors can be sustained after removing external interventions is worthy of further study.

Studies on health behavior change have typically relied on single theoretical frameworks, such as the Theory of Planned Behavior or the Protection Motivation Theory, often overlooking social and environmental factors and behavioral continuity [17]. These approaches struggle to fully explain the complexity of maintaining health behaviors. In contrast, the Multi-Theory Model (MTM) proposed by Sharma integrates behavioral intention, practical skills, and environmental changes to predict the dynamic maintenance of health behaviors [18,19]. The Protective Action Decision Model (PADM) proposed by Lindell and Perry [20] emphasizes the interaction between risk perception, resource assessment, and adaptive decision-making, which is suitable for analyzing postcrisis behavior adaptation [21]. The MTM helps understand the impact of an individual’s emotional and behavioral reactions on behavior maintenance, while the PADM focuses on behaviors in risk environments and complements the perceived factors not addressed in the MTM. The combination of these 2 models can provide a more comprehensive analytical framework for postpandemic health behavior study.

In conclusion, maintaining health behaviors is crucial for enhancing individual well-being amid the pandemic-driven public health challenges, and these challenges also provide a natural opportunity for us to investigate the maintenance of public health behaviors after the pandemic. However, public health behaviors may change due to personal psychological perceptions and external social factors after the pandemic, a topic that has not been systematically and comprehensively explored. Therefore, this study aims to investigate the status of public health behavior maintenance in the postpandemic era. By integrating the MTM and the PADM, we seek to uncover the key factors and mechanisms that influence the maintenance of public health behaviors. This study stands to enrich health behavior knowledge, and the strategies and recommendations proposed based on the findings may offer a theoretical foundation for policy implementation, inform effective public health interventions, and help build a more resilient and health-conscious society after the pandemic.

## Methods

### Framework of the Study

**Figure 1.**
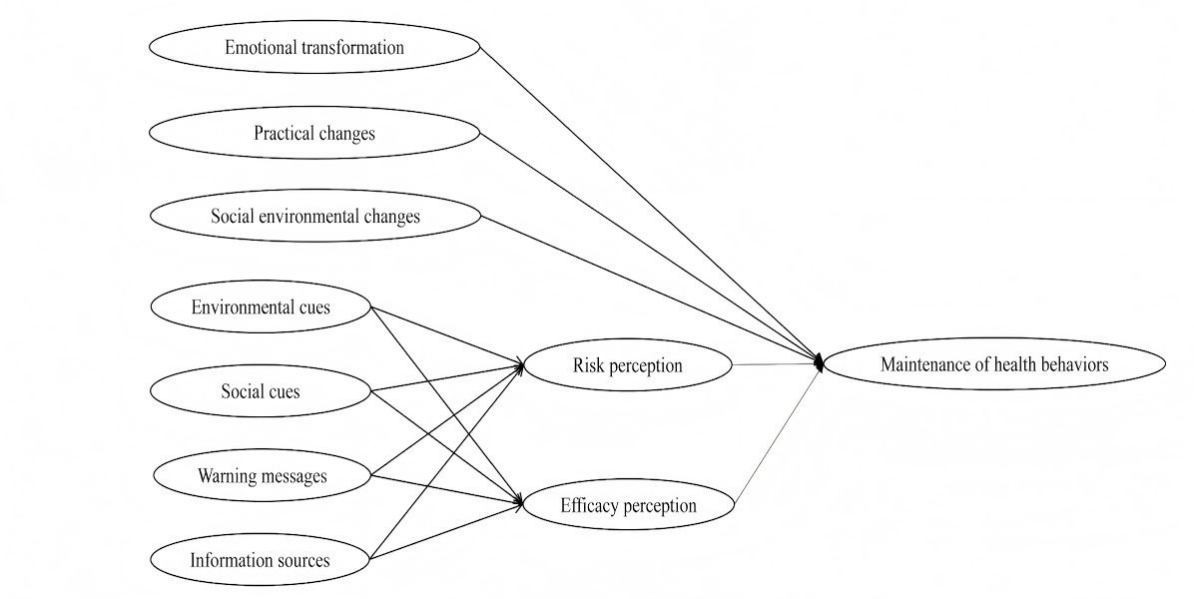
Framework of predictors for public maintenance of health behaviors based on the MTM and the PADM. MTM: Multi-Theory Model; PADM: Protective Action Decision Model.

In reference to the study by Atanasova et al [22], this study combined 2 models to explore the key factors and mechanisms influencing the maintenance of public health behaviors after the COVID-19 pandemic. Based on the previous context, these 2 models are the MTM and the PADM, both widely used to explain and predict behaviors, and in our case, we target public maintenance of health behaviors. The MTM posits that 3 factors—emotional transformation, practical changes, and social environmental changes—influence long-term behavioral maintenance [18]. Meanwhile, the PADM emphasizes that environmental cues, social cues, warning messages, and information sources trigger core perceptions, including risk perception and efficacy perception, driving protective behavioral responses [20]. In the context of health behavior maintenance after the pandemic, the MTM helps predict the continuation of these behaviors, while the PADM aids in understanding how people adapt their decisions in response to evolving risks and available resources. Combining these 2 models offers a comprehensive analysis of the factors influencing behavior maintenance from cognitive, motivational, and environmental perspectives as pandemic risks decline (Figure 1).

### Study Setting and Participants

A cross-sectional survey was conducted. From October to November 2023, we recruited participants from the general population in China using convenience sampling. The survey was conducted anonymously via the Sojump platform, a widely used online survey tool in China with a national user base. The eligibility criteria for participants were (1) voluntary completion of the questionnaire and informed consent to participate and (2) proficiency in using electronic devices.

The sample size was calculated using a formula guided by the Cochran technique [23], which is effective for large-population studies [24]. The calculation variables were n (sample size), *z*=1.96 (for a 95% confidence level), *p*=50% (the probability of selecting a choice, suitable for highly dispersed population), *q*=(1*–p*), and *d*=3.272% (allowed error percentage). According to the calculation, a sample size of 898 was determined.


$$n=\frac{z^{2}pq}{d}=\frac{1.96^{2}\times 0.5\times \left(1-0.5\right)}{0.03272^{2}}=897.0684\approx 898$$

### Recruitment Procedures

We distributed the survey links and recruitment advertisements on social media via our research team members, using the Sojump to provide survey links and widely invite eligible participants who met the inclusion criteria. To ensure data quality, all questionnaire items were set as mandatory, and each IP address, device, and account was restricted to one submission only. Also, a time limit was imposed to exclude respondents finishing the survey in less than 2 minutes. To encourage participation, those who successfully completed the survey were rewarded with 3‐5 RMB (US$ 0.42-0.70). Ultimately, 1216 valid data were obtained.

### Measurement Instruments

#### Dependent Variable

In consideration of the fact that there is currently no mature scale for assessing the maintenance of health behaviors, we adopted the Health Lifestyle and Personal Control Questionnaire developed by Darviri et al [25] to evaluate the public maintenance of health behaviors. This questionnaire comprises 26 items across 5 dimensions: healthy eating behaviors, unhealthy eating behaviors, daily life behaviors, organized physical exercise, and social and psychological balance. Respondents self-reported their sustained engagement in these behaviors during and after the COVID-19 pandemic. The scoring was as follows: “1” indicated the lowest level of maintenance (health behaviors never taken), “2” indicated a lower level of maintenance (behaviors adopted less frequently after the pandemic), “3” indicated unchanged maintenance, and “4” indicated a significant improvement in maintenance (behaviors adopted more frequently after the pandemic). Confirmatory factor analysis (CFA) indicated that the standardized factor loadings for each dimension ranged from 0.684 to 0.772, except for the second dimension (unhealthy eating behaviors) at −0.228. However, as eating behavior is also an important aspect of health behaviors, this dimension was retained. The Cronbach’s alpha (*α*=0.89) of this questionnaire demonstrated good internal consistency.

#### Independent Variables

Referring to the study by Li and Wang [26], environmental cues were assessed using 4 items with “yes” or “no” responses, scored as 1 for “no” and 2 for “yes.” A higher score indicated a stronger public perception of external environmental cues.

Based on Saghafi-Asl et al’s study [27], social cues were evaluated using 5 items rated on a 5-point scale from 1 (strongly disagree) to 5 (strongly agree). A higher score suggested that the public received more social cues about maintenance of health behaviors. These items showed good internal consistency (*α*=0.88) with standardized factor loadings ranging from 0.596 to 0.773.

Following Sun et al [28] and Wang et al [29], information sources were measured with 8 items on a 5-point Likert scale (1=never, 2=rarely, 3=sometimes, 4=often, 5=very often). A higher score indicated that the public would obtain more information about health behaviors. The CFA revealed standardized factor loadings between 0.662 and 0.775, and Cronbach’s *α*=0.86 demonstrated good internal consistency.

Warning messages were assessed through 3 items on the same 5-point Likert scale as for information sources, which were developed by Guo et al [21]. A higher score implied that the public received more information prompting them to engage in health behaviors. The CFA showed good validity (standardized factor loadings ranged from 0.837 to 0.853), and the Cronbach’s *α*=0.83 demonstrated good internal consistency.

Referring to the study by Liu et al [30], risk perception was measured with 4 items. A higher score denoted a greater perceived risk associated with unhealthy behaviors. The CFA revealed standardized factor loadings from 0.781 to 0.832. The Cronbach’s *α*=0.81 indicated good internal consistency.

Efficacy perception was assessed using a revised 5-item scale based on the study by Witte [31]. A higher score suggested a greater perception of the benefits of maintaining health behaviors. The CFA showed standardized factor loadings between 0.805 and 0.882, and Cronbach’s *α*=0.85 demonstrated good internal consistency.

According to the study by Sharma et al [19], emotional transformation, practical changes, and social environmental changes in the MTM were evaluated using 3, 2, and 3 items, respectively. Higher scores in each variable indicated a greater likelihood of maintaining health behaviors. The CFA revealed standardized factor loadings ranging from 0.746 to 0.800 for emotional transformation, 0.784 to 0.832 for practical changes, and 0.709 to 0.767 for social environmental changes. The Cronbach’s *α* values 0.81, 0.79, 0.78, respectively demonstrated acceptable internal consistency. These measurements, based on 5 variables, were consistent with the 5-point Likert scale used for social cues.

#### Control Variables

The maintenance of health behaviors is influenced by multiple factors. Based on previous literature review, we have summarized the following additional factors related to the public that may influence health behavior maintenance: (1) sociodemographic characteristics: gender, age, education, career, average monthly income, settlement type, marital status, and living condition. (2) Personal disease and health characteristics: whether they have a chronic disease, times that they have been infected with the COVID-19, self-assessment of health status, and whether they are more concerned about health after the COVID-19 pandemic.

### Statistical Analysis

The data analysis was performed using R Studio (R 4.2.3; R Core Team). The CFA was used to test the validity of the measurements, with standardized factor loadings and average variance extracted as indicators of convergent validity. The reliability of the measurements was examined using Cronbach’s α and composite reliability. Results of validity and reliability of measurements are shown in the Multimedia Appendix 1. Categorical variables were presented as frequency and proportion, and continuous variables were expressed as mean and SD. In the univariate analysis, normally distributed data were analyzed using *t* tests or ANOVA, and non-normally distributed data were analyzed using Wilcoxon rank-sum tests or rank correlation tests. Spearman correlation analysis was conducted to explore relationships between variables. Multivariate regression analysis was applied to examine impacts of demographic characteristics and key factors on the maintenance level of health behaviors. Structural equation modeling (SEM) was used to analyze pathways of factors influencing the public maintenance of health behaviors. A 2-sided *P* value below .05 was considered statistically significant.

#### Ethical Considerations

During the data collection, it was clearly stated to ensure that the survey is anonymous and all personal information would remain confidential. Participants were informed that their involvement is voluntary and they can withdraw from the study at any time. This study was approved by the Ethics Committee of Southern Medical University in Guangzhou (approval number: [2022] no. 65) and was conducted in accordance with the ethical principles outlined in the World Medical Association’s Declaration of Helsinki for medical research involving human subjects. Participants who successfully completed the survey were rewarded with 3‐5 RMB (US $0.42–0.70).

## Results

### Characteristics of Participants

A total of 726 females and 490 males participated in this survey, with an average age of 27.38 (SD 8.52) years. Among the participants, 12.83% (156/1216) reported having a chronic disease, and most of them had been infected with COVID-19 at least once (1054/1216, 86.68%). Over half rated their health as good (746/1216, 61.35%) and 73.27% (891/1216) indicated an increased concern for their health after the COVID-19 pandemic. Details are presented in Table 1.

**Table 1.** Characteristics of participants and univariate analysis of public maintenance of health behaviors.

Variables	Values, n (%)	Maintenance of health behaviors, mean (SD)	*F* test (*df*)	*P* value
Sex			0.05 (1214)	.819
Male	490 (40.30)	2.88 (0.43)		
Female	726 (59.70)	2.87 (0.47)		
Age groups (years)			46.01 (1193)^a^	<.001
<21	112 (9.21)	2.83 (0.47)		
21-31 (excluding 31)	836 (68.75)	2.84 (0.45)		
31-41 (excluding 41)	156 (12.83)	3.09 (0.42)		
≥41	93 (7.65)	2.90 (0.42)		
Education			64.12 (1211)^a^	<.001
Junior high school and below	31 (2.55)	2.78 (0.43)		
High school or junior college	83 (6.83)	2.94 (0.47)		
Three-year college	171 (14.06)	3.00 (0.52)		
Undergraduate	658 (54.11)	2.91 (0.43)		
Graduate students and above	273 (22.45)	2.72 (0.41)		
Career			61.34 (1208)^a^	<.001
Party and government officials	26 (2.14)	2.82 (0.36)		
Career employees	188 (15.46)	2.94 (0.39)		
Workers in enterprises	319 (26.23)	2.99 (0.43)		
Agricultural, forestry, livestock, or fishing water resource management personnel	39 (3.21)	2.94 (0.39)		
Self-employed	142 (11.68)	2.89 (0.50)		
Students	454 (37.34)	2.77 (0.44)		
Military personnel	2 (0.16)	3.48 (0.41)		
Others	46 (3.78)	2.79 (0.53)		
Average monthly income (yuan)^b^			91.84 (1210)^a^	<.001
<3000	451 (37.09)	2.74 (0.43)		
3000-5000 (excluding 5000)	208 (17.11)	2.87 (0.45)		
5000-8000 (excluding 8000)	312 (25.66)	3.00 (0.44)		
8000-10,000 (excluding 10,000)	145 (11.92)	3.01 (0.41)		
≥ 10,000	99 (8.14)	2.91 (0.47)		
Settlement type			0.65 (1214)	<.421
Urban	943 (77.55)	2.88 (0.45)		
Rural	273 (22.45)	2.86 (0.46)		
Marital status			85.57 (1211)^a^	<.001
Unmarried	833 (68.50)	2.80 (0.44)		
Married	374 (30.76)	3.05 (0.43)		
Divorcee	6 (0.49)	2.74 (0.52)		
Widowhood	2 (0.16)	2.81 (0.11)		
Living condition			8.53 (1214)^c^	.003
Living alone	346 (28.45)	2.82 (0.43)		
Not living alone	870 (71.55)	2.90 (0.46)		
Have a chronic disease			4.78 (1214)^d^	.029
No	1060 (87.17)	2.87 (0.45)		
Yes	156 (12.83)	2.93 (0.47)		
Times infected with COVID-19			4.54 (1212)	.209
Unknown	58 (4.77)	2.76 (0.50)		
No	104 (8.55)	2.87 (0.46)		
Once	750 (61.68)	2.89 (0.45)		
Twice or more	304 (25)	2.86 (0.44)		
Self-assessed health condition			59.01 (1211)^a^	<.001
Very poor	8 (0.66)	2.58 (0.57)		
Poor	59 (4.85)	2.77 (0.51)		
Acceptable	403 (33.14)	2.78 (0.44)		
Good	555 (45.64)	2.90 (0.42)		
Very good	191 (15.71)	3.05 (0.46)		
Concerned more about health after the COVID-19 pandemic			226.02 (1209)^a^	<.001
Strongly disagree	17 (1.40)	2.45 (0.50)		
Disagree	31 (2.55)	2.70 (0.46)		
Undecided	275 (22.62)	2.63 (0.41)		
Agree	551 (45.31)	2.86 (0.41)		
Strongly agree	340 (27.96)	3.14 (0.39)		

a *P*<.001

b 1 yuan= US $0.14.

c *P*<.01

d *P*<.05

### Univariate Analysis and Correlation Among Variables

For all participants, the overall maintenance score of health behaviors was 2.88 (SD 0.45) (Table 2). Factors such as age, education, career, average monthly income, marital status, living condition, whether one has a chronic illness, self-assessed health status, and the level of attention to personal health after the COVID-19 pandemic showed statistically significant associations with the public maintenance of health behaviors. Specifically, the factors of settlement type and time of COVID-19 infection showed no statistical differences with the public maintenance of health behaviors (Table 1). The results of the correlation analysis between variables reveal that all correlation coefficients were statistically significant, and the specific values of the correlation coefficients are shown in Table 3.

**Table 2.** Public maintenance level of health behaviors.

Dimension	Mean (SD)	
Maintenance of health behaviors	2.88 (0.45)	
Maintenance of healthy eating behaviors	2.86 (0.70)	
Maintenance of unhealthy eating behaviors	2.59 (0.73)	
Maintenance of daily life behaviors	2.97 (0.60)	
Maintenance of organized physical exercise	2.92 (0.93)	
Maintenance of social and psychological balance	2.98 (0.59)	

**Table 3.** Correlations of all latent variables.

	Maintenance of health behaviors	Environmental cues	Information sources	Warning messages	Social cues	Emotional transformation	Practical changes	Social environmental changes	Risk perception	Efficacy perception
Maintenance of health behaviors	1.00									
Environmental cues	0.11^a^	1.00								
Information sources	0.48^a^	0.19^a^	1.00							
Warning messages	0.43^a^	0.15^a^	0.67^a^	1.00						
Social cues	0.49^a^	0.10^a^	0.55^a^	0.49^a^	1.00					
Emotional transformation	0.56^a^	0.09 (*P*=.001)^b^	0.49^a^	0.45^a^	0.64^a^	1.00				
Practical changes	0.58^a^	^0.11a^	0.47^a^	0.42^a^	0.57^a^	0.73^a^	1.00			
Environmental changes	0.50^a^	0.09 (*P*=.003)^b^	0.44^a^	0.42^a^	0.56^a^	0.67^a^	0.64^a^	1.00		
Risk perception	0.32^a^	0.06 (*P*=.043)^c^	0.31^a^	0.27^a^	0.53^a^	0.48^a^	0.35^a^	0.47^a^	1.00	
Efficacy perception	0.56^a^	0.08 (*P*=.005)^b^	0.49^a^	0.41^a^	0.62^a^	0.67^a^	0.69^a^	0.62^a^	0.44^a^	1.00

a *P*<.001

b *P*<.01

c *P*<.05

### Multivariate Regression Analysis

The demographic factors found to be statistically significant in the univariate analysis, along with core study variables, were sequentially incorporated into the multivariate regression analysis using a stepwise forward method. Specifically, the inclusion order prioritized (1) statistically significant demographic factors identified in univariate analysis, (2) variables from the MTM, and (3) variables from the PADM. By the way, 2-tailed *t* test for a regression coefficient was conducted and the degrees of freedom was 1175. The results indicated that average monthly income, marital status, increased health concern after the COVID-19, emotional transformation, practical changes, information sources, warning messages, and efficacy perception influenced the public maintenance of health behaviors, with an adjusted *R^2^* of 0.455 (Table 4).

**Table 4.** Multivariate regression analysis on factors influencing the maintenance of public health behaviors (after full adjustment).

Influencing factors (control condition)	Estimate	SE	*t* test (*df*), 2-tailed	*P* value
Intercept	16.42	5.17	3.18 (1175)^b^	.002
Age, years (<21)				
21-31 (excluding 31)	–0.48	1.02	–0.47 (1175)	.639
31-41 (excluding 41)	0.47	1.35	0.35 (1175)	.725
≥41	0.87	1.58	0.55 (1175)	.581
Education (junior high school and below)				
High school or junior college	–0.53	1.92	–0.28 (1175)	.782
Three-year college	–1.27	1.83	–0.69 (1175)	.490
Undergraduate	–1.75	1.80	–0.97 (1175)	.332
Graduate students and above	–3.68	1.93	–1.90 (1175)	.057
Career (party and government officials)				
Career employees	1.09	1.89	0.58 (1175)	.562
Workers in enterprises	1.43	1.84	0.78 (1175)	.438
Agricultural, forestry, livestock, or fishing water resource management personnel	0.07	2.29	0.03 (1175)	.975
Self-employed	–0.26	1.94	–0.13 (1175)	.895
Students	2.49	2.06	1.21 (1175)	.227
Military personnel	12.23	6.50	1.88 (1175)	.060
Others	<0.01	2.25	<0.01 (1175)	.999
Average monthly income (yuan ; <3000)^c^				
3000-5000 (excluding 5000)	2.35	1.02	2.29 (1175)^d^	.022
5000-8000 (excluding 8000)	3.73	1.13	3.31 (1175)^b^	.001
8000-10,000 (excluding 10,000)	3.97	1.26	3.15 (1175)^b^	.002
≥10,000	4.07	1.34	3.04 (1175)^b^	.002
Marital status (unmarried)				
Married	1.81	0.83	2.18 (1175)^d^	.029
Divorced	1.51	3.71	0.41 (1175)	.683
Widowed	8.58	6.32	1.36 (1175)	.175
Living condition (living alone)				
Not living alone	0.75	0.64	1.18 (1175)	.237
Have a chronic disease (no)				
Yes	0.28	0.79	0.35 (1175)	.725
Self-assessed health status (very poor)				
Poor	5.06	3.36	1.51 (1175)	.132
Acceptable	3.82	3.19	1.20 (1175)	.231
Good	4.45	3.18	1.40 (1175)	.162
Very good	5.08	3.22	1.58 (1175)	.115
Concerned more about health after the COVID-19 pandemic (strongly disagree)				
Disagree	8.68	2.70	3.22 (1175)^b^	.001
Undecided	6.44	2.26	2.85 (1175)^b^	.004
Agree	8.74	2.23	3.93^a^ (1175)	<.001
Strongly agree	10.46	2.26	4.63^a^ (1175)	<.001
Emotional transformation	0.64	0.22	2.98 (1175)^b^	.003
Practical changes	0.99	0.24	4.09^a^ (1175)	<.001
Social environmental changes	0.19	0.19	1.03 (1175)	.306
Environmental cues	0.36	0.31	1.17 (1175)	.241
Information source	0.17	0.07	2.44 (1175)^b^	.015
Warning messages	0.48	0.14	3.51^a^ (1175)	<.001
Social cues	0.09	0.13	0.72 (1175)	.474
Risk perception	0.03	0.15	0.23 (1175)	.819
Efficacy perception	0.56	0.12	4.83^a^ (1175)	<.001

a *P*<.001.

b *P*<.01.

c 1 yuan=US $0.14.

d *P*<.05.

### Structural Equation Modeling and Hypothesis Testing

In the SEM, the path coefficients could reflect the strength and direction of causality between variables. Compared with regression coefficients, path coefficients capture the net effect of one variable on another after controlling variables [32]. Standardized path coefficients convert these into a unitless value for direct comparison [33]. All standardized path coefficients in this study were within the normal range, as shown in Table 5. The modified model shows good overall fit to the actual sample data, with *χ^2^*/*df* of 4.92 (*χ^2^*_353_=1737.201) and the goodness-of-fit index, comparative fit index, standardized root mean square residual, and root mean square error of approximation of 0.894, 0.923, 0.055, and 0.057, respectively. All indicators meet the requiremexcept for thet for goodness-of-fit index, which is slightly below 0.90.

**Table 5.** Path coefficients of variables in the SEM.

Hypothesis	Unstandardized coefficient	SE	*z*	*P* value	Standardized coefficient
Maintenance of health behaviors ← emotional transformation	0.296	0.045	6.635	<.001	0.213
Maintenance of health behaviors ← practical changes	0.296	0.045	6.635	<.001	0.213
Maintenance of health behaviors ← social environmental changes	0.036	0.087	0.418	.676	0.026
Maintenance of health behaviors ← efficacy perception	0.313	0.038	8.320	<.001	0.386
Maintenance of health behaviors ← risk perception	–0.095	0.038	–2.484	.013	–0.099
Risk perception ← environmental cues	0.015	0.043	0.356	.721	0.009
Risk perception ← social cues	1.130	0.081	13.939	<.001	0.789
Risk perception ← warning messages	–0.091	0.032	–2.859	.004	–0.064
Risk perception ← information sources	–0.091	0.032	–2.859	.004	–0.064
Efficacy perception ← environmental cues	–0.007	0.044	–0.149	.882	–0.003
Efficacy perception ← social cues	1.334	0.092	14.562	<.001	0.782
Efficacy perception ← warning messages	0.038	0.032	1.184	.236	0.022
Efficacy perception ← information sources	0.038	0.032	1.184	.236	0.022

The analysis shows that social cues have a positive influence on efficacy perception (*β*=.782, *P*<.001), which in turn has the most significant effect on the maintenance of public health behaviors (*β*=.386, *P*<.001). Emotional transformation and practical changes from the MTM also positively affect public health behavior maintenance (both *β*=.213, *P*<.001). Risk perception is found to have a negative impact on public health behaviors (*β*=−.099, *P*=.013). Social cues are the most influential factor for risk perception with a positive effect (*β*=.789, *P*<.001), while warning messages and information sources are negatively associated with risk perception (both *β*=−.064, *P*=.004).

## Discussion

### Principal Findings

This study found that the public maintenance of health behaviors was at a fairly low level (mean 2.88, SD 0.45) after the COVID-19 pandemic. Similar to the study of Wang and Gago [34], children exhibited health behavior maintenance issues after the pandemic, such as poor sleep, increased screen time, and changed daily activities. When examining the dimensions of health behavior maintenance, the public surveyed in this study maintained relatively stable social and psychological balance, daily life behaviors, and organized physical exercise. However, the maintenance of dietary behaviors showed significant fluctuation. This echoes the study by van Reekum et al [35], which highlighted the difficulty in sustaining dietary patterns formed during the pandemic once normal life resumed. Moreover, as the pandemic progressed, pandemic fatigue set in, leading to a decline in health behaviors due to the sustained high level of vigilance required [36]. Even a short-term decrease in health behavior maintenance may adversely affect health. Therefore, it is crucial to focus on public health behavior maintenance and its influencing factors after the pandemic and to implement effective and timely interventions.

In this study, factors such as average monthly income, marital status, and personal health concern after the COVID-19 pandemic were found to be associated with the public maintenance of health behaviors. Wambiya et al [37] similarly found that low-income groups, burdened by economic hardships and work demands, may neglect the role of health behaviors in promoting physical health. Meanwhile, spousal encouragement, support, and supervision can aid in maintaining health behaviors [38]. In addition, individuals with higher health concerns tend to have better health cognition, enabling them to practice health behaviors more effectively [39]. Notably, this study found no correlation between settlement type or whether individuals have been infected with COVID-19 and public health behavior maintenance. This is inconsistent with the study of Wollast et al [40]. The discrepancy may arise because most participants surveyed in this study had been infected with COVID-19 (86.68%). With frequent social interactions and population movements between urban and rural areas, resulting in widespread COVID-19 infections [41], which led to no significant differences in population distribution.

This study confirms that emotional transformation and practical changes in the MTM are positive predictors of health behavior maintenance, aligning with prior study [42]. Emotional transformation emphasizes redirecting negative emotions associated with health behaviors, such as reluctance and resistance, toward the goal of maintaining health behaviors [43]. Practical changes focus on identifying and correcting barriers and incorrect practices in implementing health behaviors through recording or reflection [44]. Throughout these 2 processes, individuals typically combine intrinsic self-motivation with extrinsic emotional support, seeking solutions that use both internal and external resources to overcome psychological barriers in maintaining health behaviors.

This study reveals that warning messages and information sources in the PADM negatively impact risk perception, which in turn undermines the public maintenance of health behaviors. This contradicts Cao and Liu’s [45] findings in their study on internet users. Generally, access to public health information is positively correlated with health behaviors [46]. However, in this study, the public might have encountered an abundance of conflicting and dubious health information, resulting in confusion, information overload, and distrust [47]. This aligns with Pedro et al’s [48] study, which could diminish sensitivity to health risks. Some studies point out that risk perception is a positive predictor of health behaviors [49,50], but excessive risk perception may trigger impatience or rebelliousness, decreasing individuals’ willingness and frequency of action [51]. Therefore, the bidirectional effects of information reception and risk perception on behavior warrant further exploration.

This study indicates a positive correlation between efficacy perception and the public maintenance of health behaviors, consistent with existing studies [52,53]. Individuals with stronger beliefs in their ability to maintain health behaviors exhibit higher self-control and willingness, highlighting the beneficial role of confidence and motivation in adhering to health practices [54]. Moreover, a greater perception of health behavior effects strengthens their belief in maintaining these behaviors [55]. However, this study found that information sources and warning messages do not influence health behavior maintenance through efficacy perception, conflicting with the hypothesis in the PADM [20]. This might be due to asymmetric information transmitted through these channels, leading to varying efficacy perceptions. When individuals perceive insufficient benefits, they tend to adopt a conservative behavior pattern and are less likely to persist in health behaviors [56].

This study found that social cues positively influence risk perception and efficacy perception, in turn affecting public health behavior maintenance, consistent with the PADM [20]. Individuals with greater exposure to social cues show heightened awareness of potential behavioral risks [57] and acquire more health-related information. This increased awareness and information boost their confidence in maintaining health behaviors and motivate them to sustain these behaviors. However, unlike previous studies [58,59], no statistical link was found between environmental cues and health behavior maintenance through risk and efficacy perception, nor any impact of social environmental changes on health behavior maintenance. This might be because most participants had COVID-19–related experiences during the study period. Due to frequent social interactions and population movements, people’s sensitivity to environmental cues and social environmental changes decreased compared to the pandemic period. Similar to a “gray rhino” event, individuals tend to overlook health risk warnings when they are accustomed to a situation [60].

### Strengths and Limitations

The MTM and the PADM are both useful for studying behavior change mechanisms. The MTM focuses on individual psychology and emotional shifts, while the PADM emphasizes external environmental information. Despite their different focal points, they have not been integrated into previous studies. This study combines their strengths to analyze factors influencing public health behavior maintenance. It takes into account the public real-time health risk-benefit perception and behavioral responses after the pandemic, offering a new perspective on health behavior study and providing references for promoting public health behavior maintenance.

This study offers initial insights into the health behavior maintenance and its influencing factors among the Chinese public, but it has limitations that need further exploration. The results of this study rely on self-reporting by the participants, which may be prone to recall bias. Future research could use smart wearable devices to collect objective data on health behavior maintenance, such as daily step counts and sleep duration. In addition, the online survey with convenience sampling leans toward younger participants who are more tech-savvy, while they can provide unique perspectives on this study. To enhance data representativeness, future studies can combine online and offline recruitment channels, with regional stratified sampling to recruit more diverse participants, and embrace a longitudinal design to better establish causal relationships between factors.

### Implications

Drawing on this study, we propose the following suggestions to enhance public health behavior maintenance. First, government, health departments, and medical institutions should be actively engaged as authoritative sources to promote public health behaviors, leveraging their authority and credibility. Second, integrate traditional, online, and social media to broaden information-sharing channels, innovate dissemination methods, and embed health concepts into public daily life. While propagandizing the benefits of health behaviors, it is also necessary to educate the public about the dangers of unhealthy behaviors and corresponding health warning messages. It is crucial to avoid false information and exaggerated information. Finally, enhance family and social support to help individuals manage negative emotions related to health behavior maintenance and encouraging self-motivation for effective health behavior management.

### Conclusions

This study, integrating the MTM and the PADM, explores the status and influencing mechanisms of public health behavior maintenance after the COVID-19 pandemic. Our findings indicate that enhancing emotional transformation, practical changes, social cues, and warning messages can boost health behavior maintenance. However, attention should be given to pandemic-related fatigue, and the risk information should be communicated properly. Also, external support should be integrated to boost the public’s sense of efficacy in maintaining health behaviors. This study attempts to integrate the MTM and the PADM within the Chinese cultural context and offers perspectives on health behavior studies. It provides some strategies and recommendations for promoting the public maintenance of health behaviors.

## Supplementary material

10.2196/66535 Multimedia Appendix 1 Validity and reliability of measurements.

10.2196/66535 Checklist 1 STROBE Checklist for reporting cross-sectional studies.

## Abbreviations

CFA: confirmatory factor analysis
MTM: Multi-Theory Model
PADM: Protective Action Decision Model
SEM: structural equation modeling
