# Supplementary material for: Factors Influencing the Maintenance of Public Health Behaviors After an Epidemic: Cross-Sectional Study
Source: JMIR Public Health Surveill. 2025 Jul 23;11:e66535. doi: 10.2196/66535 (PMC12309420; doi:10.2196/66535)
Supplement: Multimedia Appendix 1 [file publichealth-v11-e66535-s001.docx]

Table S1.

| Standardized  factor *Z* Cronbach α | | | | | CR | AVE |
| --- | --- | --- | --- | --- | --- | --- |
|  | loadings |  |  |  |  |  |
| mhb1 | 0.762 | 0.026 | 29.483*** |  |  |  |
| Maintenance mhb2 | -0.228 | 0.030 | -7.483*** |  |  |  |
| of health mhb3 | 0.772 | 0.026 | 30.032*** | 0.891 | 0.737 | 0.457 |
| behaviors mhb4 | 0.684 | 0.027 | 25.469*** |  |  |  |
| mhb5 | 0.766 | 0.026 | 29.677*** |  |  |  |
| in_so1 | 0.775 | 0.026 | 29.829*** |  |  |  |
| Information  in_so2 | 0.662 | 0.027 | 24.240*** | 0.856 | 0.772 | 0.532 |
| sources  in_so3 | 0.745 | 0.026 | 28.296*** |  |  |  |
| wa_me1 | 0.840 | 0.024 | 34.811*** |  |  |  |
| Warning  wa_me2 | 0.853 | 0.024 | 35.616*** | 0.826 | 0.881 | 0.712 |
| messages  wa_me3 | 0.837 | 0.024 | 34.621*** |  |  |  |
| so_cu1 | 0.677 | 0.027 | 25.329*** |  |  |  |
| so_cu2 | 0.596 | 0.028 | 21.576*** |  |  |  |
| Social cues so_cu3 | 0.716 | 0.026 | 27.287*** | 0.881 | 0.828 | 0.492 |
| so_cu4 | 0.773 | 0.025 | 30.349*** |  |  |  |
| so_cu5 | 0.731 | 0.026 | 28.073*** |  |  |  |

Latent variables

Observed variables

Standard error

perception

| re | 0.805 | 0.025 | 32.212*** | 0.848 0.833 0.714 | | |
| --- | --- | --- | --- | --- | --- | --- |
| et1 | 0.746 | 0.025 | 29.292*** |  |  |  |
| et2 | 0.760 | 0.025 | 30.029*** | 0.812 | 0.813 | 0.592 |
| et3 | 0.800 | 0.025 | 32.303*** |  |  |  |

| Risk | pse | 0.781 | 0.027 | 28.548*** |
| --- | --- | --- | --- | --- |
| perception | psu | 0.832 | 0.027 | 30.645*** |
| Efficacy | se | 0.882 | 0.024 | 36.506*** |

Emotional transformation

0.805 0.789 0.651

| Practical  changes | pc1  pc2 | 0.784  0.832 | 0.025  0.025 | 31.134***  33.701*** | 0.790 0.791 0.654 | | |
| --- | --- | --- | --- | --- | --- | --- | --- |
| Social | sec1 | 0.749 | 0.026 | 28.528*** |  |  |  |
| environmental | sec2 | 0.767 | 0.026 | 29.481*** | 0.784 | 0.786 | 0.551 |
| changes | sec3 | 0.709 | 0.027 | 26.526*** |  |  |  |

Note: ^***^: *P*<.001, mhb: maintenance of health behaviors, in_so: information sources, wa_me: warning messages, so_cu: social cues, pse: perceived severity, psu: perceived susceptibility, se: self efficacy, re: response efficacy, et: emotional transformation, pc: practical changes, sec: social environmental changes, CR: composite reliability, AVE: average variance extracted.
